# Bridging the Chemical Profile and Biological Activities of a New Variety of *Agastache foeniculum* (Pursh) Kuntze Extracts and Essential Oil

**DOI:** 10.3390/ijms24010828

**Published:** 2023-01-03

**Authors:** Fănică Bălănescu, Andreea Veronica Botezatu, Fernanda Marques, Anna Busuioc, Olivian Marincaş, Costel Vînătoru, Geta Cârâc, Bianca Furdui, Rodica Mihaela Dinica

**Affiliations:** 1 Faculty of Medicine and Pharmacy, “Dunărea de Jos” University of Galati, 35 A.I. Cuza Street, 800010 Galati, Romania; 2 Faculty of Sciences and Environment, Department of Chemistry Physical and Environment, “Dunărea de Jos” University of Galati, 111 Domnească Street, 800201 Galati, Romania; 3 Departamento de Engenharia e Ciências Nucleares (DECN), Centro de Ciências e Tecnologias Nucleares, Instituto Superior Técnico, University of Lisbon, Campus Tecnológico e Nuclear, Estrada Nacional 10, Km 139.7, Bobadela, 2695-066 Boticas, Portugal; 4 National Institute for Research and Development of Isotopic and Molecular Technologies, 67-103 Donat Street, 400293 Cluj-Napoca, Romania; 5 Plant Genetic Resources Bank for Vegetables, Floriculture, Aromatic and Medicinal Plants Buzău, 56 Nicolae Bălcescu Street, 120187 Buzau, Romania

**Keywords:** *Agastache foeniculum*, xanthine oxidase inhibitors, antioxidants, bioactive compounds

## Abstract

This study investigated the phytochemical content of alcoholic extracts and essential oil of a new variety of medicinal plants, *Agastache foeniculum* (Pursh), which Kuntze adapted for cultivation in Romania, namely “Aromat de Buzău”. The essential oil was investigated by GC-MS, while the identification and quantification of various compounds from alcoholic extracts were performed by HPLC-DAD. The total phenol and flavonoid contents of the extracts were evaluated by using standard phytochemical methods. The antioxidant activities of ethanol, methanol extracts, and essential oil of the plant were also assessed against 2,2′-diphenyl-1-picrylhydrazyl (DPPH^•^), 2,2′-azino-bis-(3-ethylbenzothiazoline-6-sulphonic acid) (ABTS^•+^), and by ferric reducing power (FRAP) using spectroscopic methods. Cyclic voltammetry was used to evaluate the antioxidant capacity of the essential oil. The concentrations of phenolic compounds were higher in methanolic extract compared to ethanolic extract. A significant correlation was found between total phenol and total flavonoid contents (r = 0.9087). Significant high correlations were also found between the total phenolic compounds and the antioxidant activities of the extracts (r ≥ 0.8600, *p* < 0.05). In addition, the extracts and essential oil showed good antioxidant and xanthine oxidase inhibitory activities. Estragole was detected as the major constituent of the essential oil (94.89%). The cytotoxic activity of the essential oil was evaluated by the MTT assay. At lower concentrations (1 µg/mL) high cytotoxicity against MCF-7 breast cancer cells was observed but not on the non-tumoral dermal fibroblasts (HDF) which indicated selectivity for cancer cells and suggests the presence of biologically active components that contribute to the observed high cytotoxic effect. Findings from the present study offer new perspectives on the use of *A. foeniculum* as a potential source of bioactive compounds and a good candidate for pharmaceutical plant-based products.

## 1. Introduction

Several studies have associated many diseases such as cancer or gout with increased oxidative stress, resulting from an imbalance between reactive oxygen species (ROS) and antioxidants [1]. Cancer is considered the second cause of death worldwide, after ischemic heart disease and stroke, according to the world health organization (WHO) statistics on global health [2]. Oxidative stress, by inducing gene mutations and activating pro-oncogenic signaling, can generate carcinogenesis and the progression of cancer cells [3]. In the treatment of several diseases, the aim is to reduce the accumulation of ROS that occurs as a result of metabolic disorders [1,4,5,6]. Inhibition of xanthine oxidase (XO) reduces both vascular oxidative stress and circulating uric acid levels, which helps reduce the risk of gout [7]. XO inhibitors (XOIs) work by blocking the biosynthesis of uric acid from purines in the body and can act either at the purine binding site such as allopurinol or at the cofactor site like benzimidazole [8,9]. Known XO inhibitors such as allopurinol, oxypurinol, and febuxostat have been widely used for the treatment of hyperuricemia and gout [7]. However, due to their side effects, the identification of new non-purine selective xanthine oxidase inhibitors is sought [10].

Over the centuries, empirical knowledge about the benefits of medicinal plants in alternative medicine has been passed from generation to generation [11,12]. The health benefits of chemical compounds from natural sources have gained a growing interest in modern medicine. More and more herbal products are used as sources of bioactive compounds, such as in herbal teas, food supplements with herbal powders, essential oils (EOs), extracts obtained in different solvents, purified isolated organic compounds, and in synergistic drug combinations consisting of classic target synthetic molecules with compounds from natural sources [13,14]. In the food industry, antioxidants are added to food, in order to prevent the deterioration of its taste, smell, and color [15]. Previous studies have reported that the consumption of synthetic antioxidants was related to possible adverse effects such as skin allergies, gastrointestinal tract problems, and increased risk of cancer [15]. Therefore, it is necessary to find compounds from natural sources with therapeutic action to circumvent the severe side effects of synthetic drugs [16]. The use of compounds from natural sources has many advantages, such as chemical diversity, targeting multiple host sites through various mechanisms, displaying high biological specificity, and low side effects [17,18].

Many plants of the *Lamiaceae* family are used in traditional medicine as herbal products, but also in the food industry as flavorings [19,20]. The genus *Agastache* is part of the *Lamiaceae* family and includes 22 species of perennial ornamental and medicinal plants [21]. *Agastache foeniculum* (Pursh) Kuntze with fragrant leaves and purple flowers is widely used for ornamental purposes, flavoring sweets, and other foods due to its appearance and pleasant aromas similar to anise [22]. This plant is used to produce herbal teas that are especially preferred by native Americans for therapeutic purposes such as relieving various symptoms of colds, fevers, coughs, heart disease, inflammation, and pain [23]. This herbaceous and aromatic perennial plant is native to North America [24] and was mentioned in other studies as anise hyssop, *Lophantus anisatus*, blue giant hyssop, flagrant giant hyssop, or lavender giant hyssop [22,25,26].

So far, in the literature, several studies have represented *A. foeniculum* phytochemical content and its pharmacological properties. The phytochemical profile of these plants includes non-volatile metabolites belonging to several classes such as flavones and flavone-glycosides (a rare dimeric malonylflavone—agastachin, agastachoside, acacetin, apigenin, tilianin, myricetin, luteolin) [22,27], phenolic compounds (rosmarinic acid, caffeic acid) [28], lignans (agastenol, agastinol) [29], terpenoids including triterpenoids (betulin, betulinic acid, maslinic acid, oleanolic acid, β-amyrin, ursolic acid, corosolic acid, α-amyrin), diterpenes (agastaquinone, agastol), and sterols [22,30,31]. Volatile metabolites were reported in their chemical composition such as estragole, pulegone, eugenol, methyleugenol, menthone, isomenthone, and spathulenol [20,30,32].

In the current context, taking into account the worldwide largest number of diseases that affect humans such as gout, cardiovascular diseases, diabetes or cancer [2,33,34,35], the adapted cultivation of various plants from other geographical regions is very important to properly reduce the costs of cultivating, processing, and transport, and also to make more plant products with therapeutic applications [36] available. This innovative, economical, and sustainable approach is also able to promote the impact of natural compounds from plants on human health. Based on the antioxidant potential of the main components identified in the EO and extracts from *A. foeniculum*, this paper aimed to evaluate the phytochemical characterization of a new Romanian plant variety *A. foeniculum* (Pursh) Kuntze, namely “Aromat de Buzau” (AdB) registered in 2018 (https://istis.ro/en/catalog-oficial/, accessed on 15 September 2022), which has been adapted to the climatic cultivation conditions in Romania by the Research-Development Station for Vegetable Growing Buzău. Moreover, this study sought to evaluate the biological properties of the alcoholic extracts and EO from this plant, such as antioxidant, xanthine oxidase inhibitory, and cytotoxic activities.

## 2. Results

### 2.1. Morphological and Structural Characteristics of A. foeniculum AdB Using Confocal Laser Scanning Microscopy (CLSM)

**Figure 1:**
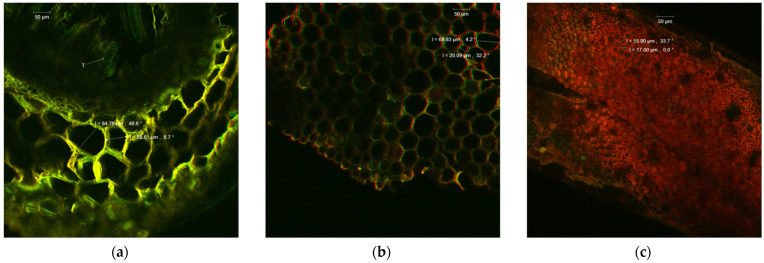
Sections of (**a**,**b**) stem and (**c**) leaves of *A. foeniculum* AdB observed using CLSM.

The present study shows morphological and structural characteristics of the leaves and stems of a plant adapted to cultivation in Romania by confocal laser scanning microscopy. The sections through the stems and leaves of the mature plant of the new variety of *A. foeniculum* AdB indicated healthy plant tissues, with normal morphological and structural characteristics, as observed by confocal laser scanning microscopy (Figure 1). Microscopic images of fresh cross-sectional samples through the *A. foeniculum* stem showed cortical parenchyma under the epidermis, with large polygonal cells (58.61–84.78 µm). The medullary area was rich in vascular elements (T) with a thick secondary wall with spiral ornaments. The thick secondary cell wall is important in the transport of organic compounds synthesized in the assimilating parenchyma (Figure 1a,b). The leaf section of *A. foeniculum* presents a very compact assimilating tissue with small intercellular spaces, polygonal cells of approximately 15 ± 2 µm, and thick cell walls (Figure 1c).

### 2.2. Profiling of Chemical Compounds in A. foeniculum AdB EO by GC-MS

Hydrodistillation of the A. foeniculum AdB plant gave an odorous pale yellow EO with a yield of 1.86 ± 0.64% (*v/w*), considering three independent extraction procedures (*n* = 3).

**Figure 2:**
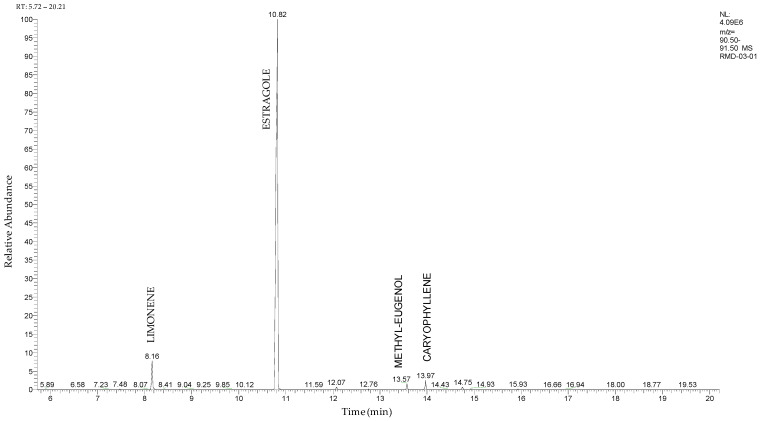
Total ion chromatogram of the EO from *A. foeniculum* AdB using GC–MS.

The total ion chromatogram recorded by GC–MS of the EO from *A. foeniculum*, which contained seven identified compounds, is shown in Figure 2, and the chemical composition is presented in Table 1.

The main components detected in *A. foeniculum* were estragole, limonene, methyl eugenol, and caryophyllene (Figures S1–S8), together with other volatile compounds (Table 1). Previous investigations on *A. foeniculum* revealed different chemical compositions of the EOs in which the major compounds detected were methylchavicol, β-caryophyllene, limonene, menthone, linalool, silvestrene, or thymol [22,37]. Therefore, there is diversity in the chemical composition of *Agastache* species, which is mainly reflected in the EOs and biological activities due to several environmental, cultivation, and harvesting factors.

### 2.3. Total Phenolic and Flavonoid Contents

By ultrasound-assisted extraction, methanolic extracts were obtained with a yield of 11.062 ± 0.945% and ethanolic extracts with a yield of 7.212 ± 0.686%, considering three independent extractions. Total phenolic (TPC) and flavonoid (TFC) contents were determined both for methanolic (MeOH) and ethanolic (EtOH) extracts from the dried stem, flowers, and leaves of *A. foeniculum*. Results are summarized in Table 2. The methanolic extract was characterized by the highest phenolic and flavonoid contents.

### 2.4. Identification of Bioactive Compounds in the Extracts from A. foeniculum AdB through HPLC-DAD

**Figure 3:**
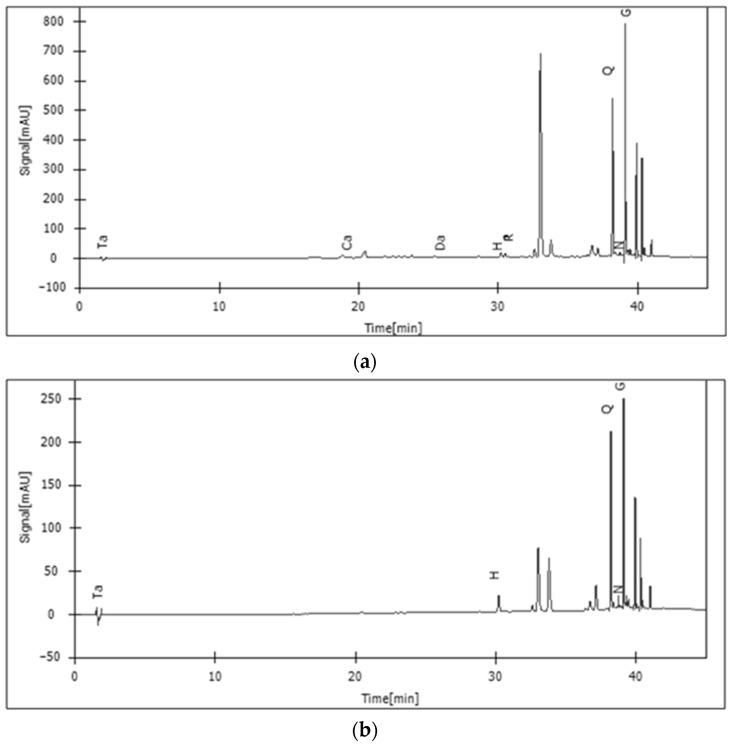
HPLC–DAD chromatograms of the samples: (**a**) *A. foeniculum*- methanolic extract (Ta—tannic acid, Ca—caffeic acid, Da—daidzein, H—hyperoside, R—rutin, Q—quercetin, N—naringenin, G—genistein); (**b**) *A. foeniculum*- ethanolic extract (Ta—tannic acid, H—hyperoside, Q—quercetin, N—naringenin, G—genistein).

In this work, the phytochemical profile of alcoholic extracts from *A. foeniculum* AdB was investigated by HPLC-DAD analysis (Figure 3). The chromatographic study of the extracts showed that the methanolic and ethanolic extracts revealed high quantities of quercetin and genistein. The quantification of each compound in the studied extracts is presented in Table 3.

In the methanolic extracts, the concentrations of all compounds detected were higher compared to ethanolic extracts. Genistein and quercetin were found in all samples tested, while tannic acid was quantifiable only in the sample extracted with methanol. Caffeic acid, *p*-coumaric acid, quercetin, hyperoside, and rutin were identified in higher concentrations in methanolic extracts.

### 2.5. In Vitro Antioxidant Activities

Free radicals are produced in biological systems and are also found as exogenous and are known to influence various chronic degenerative diseases such as cardiovascular disease, inflammatory diseases, aging, carcinogenesis, or arthritis [6,38]. Antioxidant compounds protect cells against oxidative stress through a mechanism of intervention on one of the three major stages of the oxidative process mediated by free radicals, namely initiation, propagation, and termination. These antioxidants are found naturally in many foods, and the balance between oxidants and antioxidants in the body can have a significant effect on human health [39].

#### 2.5.1. 2,2′-Diphenyl-1-Picrylhydrazyl (DPPH) Radical Scavenging Activity

**Figure 4:**
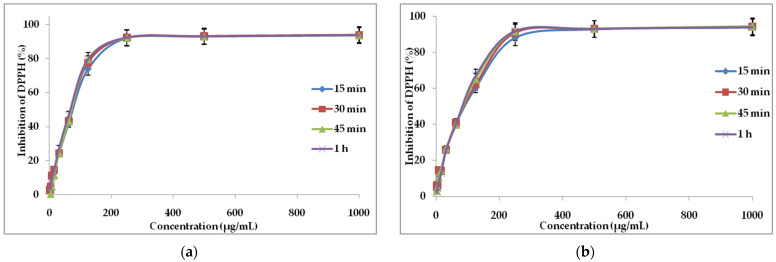

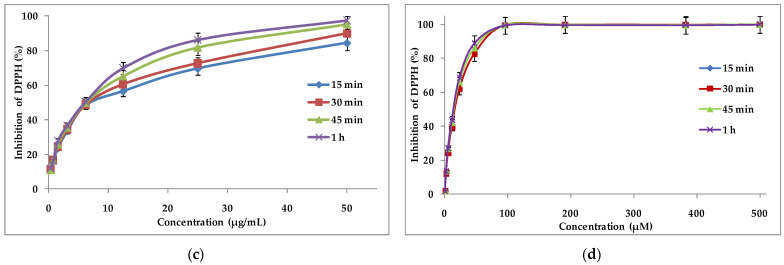
DPPH scavenging activity of (**a**) methanolic extract, (**b**) ethanolic extracts from *A. foeniculum* AdB, (**c**) EO from *A. foeniculum* AdB, and (**d**) gallic acid. Each value is the mean of three replicates ± SD.

Plants with a high content of secondary metabolites show antioxidant activity due to their redox properties and chemical structures. The MeOH extract, EtOH extract, and EO of *A. foeniculum* AdB showed strong antioxidant activity against the investigated free radicals (Figure 4). The phenolic content could justify the antioxidant activity of the alcoholic extracts from *A. foeniculum* AdB. The percentage of inhibition of DPPH radicals by the extracts did not vary significantly in time (from 15 min up to 1 h), unlike the EO where it was found that the same concentration of EO increases its DPPH radical inhibition activity in time (Figure 4c).

#### 2.5.2. ABTS [2,2′-Azinobis-(3-ethylbenzothiazoline-6-sulfonic Acid)] Scavenging Activity

**Figure 5:**
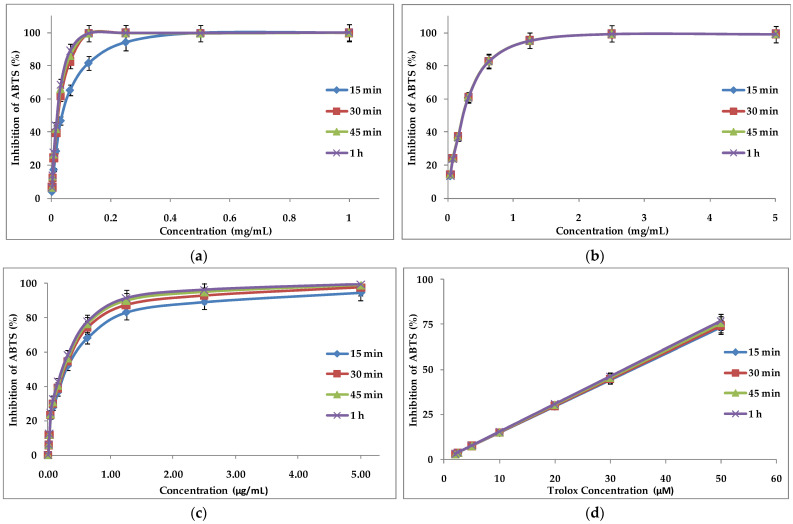
ABTS scavenging activity of (**a**) MeOH extract; (**b**) EtOH extract; (**c**) EO, from *A. foeniculum* AdB; and (**d**) Trolox. Each value is the mean of three replicates ± SD.

Powerful antioxidants prevent biomolecules (proteins, sugars, nucleic acids, polyunsaturated lipids) from suffering oxidative damage through free radical-mediated reactions. Discoloration of the ABTS solution is determined by the percentage of inhibition of the ABTS^•+^ radical cation as a function of sample concentration and time (Figure 5). These plant extracts and EO were found to have antioxidant effects against ABTS^•+^ radical cations that may justify and therefore encourage their use in medicine.

#### 2.5.3. FRAP Scavenging Activity

The assay consists of the reduction of ferric to ferrous ions at low pH, providing a colored complex. Ferric tripyridyltriazine (TPTZ 2,4,6-Tris(2-pyridyl)-s-triazine) has a yellow color that, upon reduction to ferrous form by antioxidants, turns to a violet-blue color. The change in absorbance is proportional to the total ferric-reducing power of the antioxidants in the sample. The MeOH extract demonstrated higher total ferric-reducing power (45.721 ± 0.014 µM Fe(II) equivalents/g of extract) when compared to the EtOH extract (39.483 ± 0.017 µM Fe(II) equivalents/g of extract), which indicates that the MeOH extract contains a higher amount of antioxidant compounds (Table 4).

Pearson’s correlation coefficients between the means of phytochemical contents (TPC, TFC) and antioxidant activities (DPPH^•^, ABTS^•+^, FRAP) were computed and reported in Table 5. A statistically high significant correlation (*p* < 0.01) coefficient was found between total flavonoid content and free radical scavenging activity against DPPH^•^ (r = 0.9972) and ABTS^•+^ (r = 0.9813). Correlation coefficients were also very high among the three antioxidant activity values (DPPH^•^, ABTS^•+^, FRAP) (r ≥ 0.8600).

### 2.6. Electrochemical Evaluation of Antioxidant Capacity by Cyclic Voltammetry

**Figure 6:**
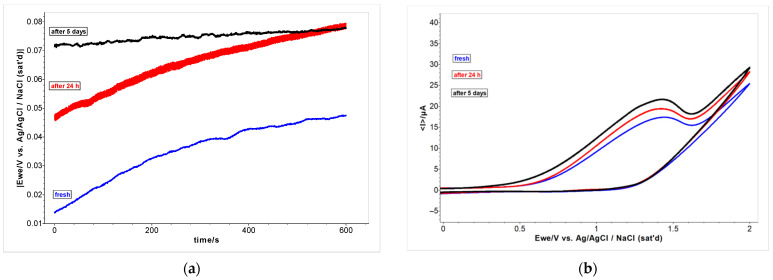
(**a**) The evolution of the potential (E) by OCV registered for 10 min of *A. foeniculum* AdB EO with time; (**b**) Cyclic voltammograms of *A. foeniculum* AdB EO registered at 24 h and 5 d (E = ± 2 V/Ag/Ag Cl_sat_, at 100 mVs^−1^).

Fresh samples of 5 µL of EO in 20 mL methanol were prepared and analyzed by open circuit voltage (OCV), after 24 h and 5 d (samples were kept in the fridge). The evolution of the potential (E) of the samples registered for 10 min can be observed in Figure 6. The potential of the sample analyzed after 24 h showed a difference of about +30 mV in the positive zone compared with the fresh sample. In the next 30 min and further, no noticeable differences were observed, but an increase in the potential was registered. Results indicate the availability of electronic exchanges between chemical components from the sample since they are systems with dynamic evolution that have an increasing value of E to 100 mV after 30 min.

**Figure 7:**
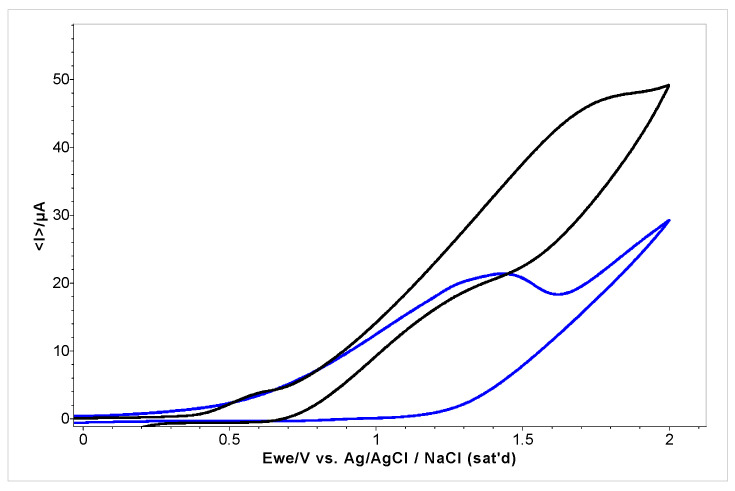
Cyclic voltammograms of *A. foeniculum* AdB EO (blue) and standard compound eugenol (black), E = ±2 V/Ag/Ag Cl_sat_, at 100 mVs^−1^.

The cyclic voltammograms of the standard compound eugenol, a natural compound with known antioxidant properties, and *A. foeniculum* EO are shown in Figure 7. In both samples, at ca. 0.5 V/Ag/AgCl_sat,_ the cyclic voltammograms showed biocompounds that improve the potential intensity. In the sample with the EO from *A. foeniculum* AdB, a maximum anodic current of 20–22 μA was registered at the potential between 1.5 till 1.9 V. The half-wave potential (E_½_) of eugenol was registered around 1.85 V/Ag/AgCl_sat_.

### 2.7. Xanthine Oxidase (Xo) Inhibitory Activity of the EO and Alcoholic Extracts from A. foeniculum

**Figure 8:**
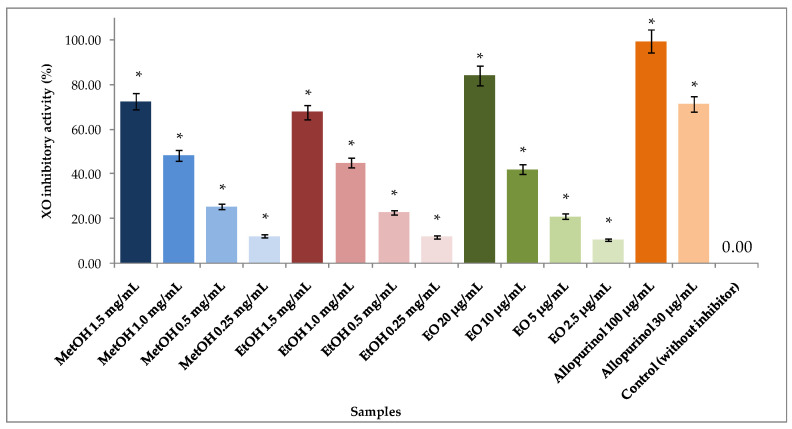
Xanthine oxidase (XO) inhibitory activity of allopurinol, extracts, and EO from *A. foeniculum* AdB evaluated at varying concentrations. Results are mean ± SD of three replicates per condition. * Significantly different (Dunnett *t*-tests, 2-sided) from the control (without inhibitor) conditions (*p* < 0.001).

Xanthine oxidase is involved in purine degradation in humans by forming xanthine from hypoxanthine and is ultimately converted to uric acid by an enzymatic reaction catalyzed by xanthine oxidase. Uric acid is eliminated in the urine, but excessive uric acid formation can consequently lead to hyperuricemia and gout [8,10]. The EO from *A. foeniculum* AdB demonstrated a high inhibition of xanthine oxidase activity in vitro (84.077 ± 0.031%) at a concentration of 20 µg/mL, which is comparable with that of 30 µg/mL allopurinol, a well-known XO inhibitor [8]. The EO and the alcoholic extracts demonstrated significant inhibitory activities at concentrations higher than 2.5 µg/mL (*p* < 0.001) and higher than 0.25 mg/mL (*p* < 0.001), respectively (Figure 8).

### 2.8. Cytotoxic Activity of the EO from A. foeniculum

**Figure 9:**
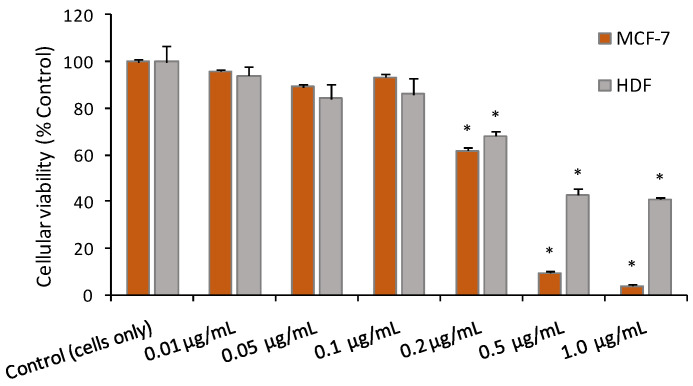
Cytotoxic activity of *A. foeniculum* EO against MCF-7 and HDF cell lines. The IC_50_ values found using the GraphPad Prism software (vs. 5.0) were MCF-7, 0.24 ± 0.09 μg/mL, and HDF, 0.47 ± 0.1 μg/mL. Control = cells without treatment. Data are mean ± SD of two independent experiments with at least four replicates per condition. * Significantly different (Dunnett *t*-tests, 2-sided) from the basal conditions (*p* < 0.001).

In the search for new medicines for treating cancer, the compounds isolated from plants could be better alternatives to the current chemotherapeutics that present poor selectivity and high toxicity, hoping to get lesser side effects and to spare healthy cells and tissues [2]. Certain components of medicinal plants have proved to be alternative approaches in the fight against cancer, either administered alone or combined with chemotherapeutic drugs to improve the treatment efficacy. As an interesting example, the cytotoxic activity of estragole from the EO from *A. foeniculum* AdB was tested against MCF-7 breast cancer cell lines [23]. As shown, estragole exhibited a dose-dependent cytotoxic effect in the MCF-7 cells showing IC_50_ values of 74 μg/mL [23]. Our studies with the EO from the Romanian *A. foeniculum* AdB and the MCF-7 cell lines indicated a higher cytotoxic effect against MCF-7 breast cancer cell lines at lower concentrations (1 µg/mL) which suggested the presence of other biologically active components that contributed to the high cytotoxic effect observed. In addition, using the normal cells, the human dermal fibroblasts, the cytotoxic effect seemed to indicate improved selectivity for the breast cancer cells, at concentrations higher than 0.2 µg/mL (Figure 9). The EO demonstrated statistically significant cytotoxic activities against MCF-7 cells at concentrations higher than 0.2 µg/mL (*p* < 0.001). Further studies will be carried out to evaluate the cytotoxicity of the alcoholic extracts.

## 3. Discussion

Human diseases are becoming more aggressive and resistant to classical drugs, therefore the demand for new treatments is increasingly aiming to explore new sources of natural multi-target drugs [40]. Plants are an increasingly explored sources in the search for new drugs. According to previous studies, the efficacy of natural compounds with antioxidant activity for therapeutic purposes was demonstrated, and future studies may expand their applications in clinical therapies [4].

This study investigated the chemical composition and biological properties of two alcoholic extracts, and EO from the *A. foeniculum* AdB variety. The sections through the stems and leaves from the mature plant adapted to cultivation in Romania observed by CLSM indicated healthy plant tissues, with normal morphological and structural characteristics. The yield of EO from *A. foeniculum* AdB is similar to other results reported in the literature [22]. The main component detected in the EO from *A. foeniculum* AdB was estragole (1-allyl-4-methoxybenzene), an allylbenzene analog, a colorless liquid with an odor of anise and sweet taste. In this study, we found a slightly different chemical composition of the EO compared to other studies, but also some common chemical components. Variations in the phytochemical composition occur due to the geo-climatic conditions of their growth, maturity at the time of collection, or species variation [36]. Charles et al. [41] reported the analysis of 19 varieties of *Agastache* species for EO content and 26 compounds were identified. The main constituents of the oil from *A. foeniculum* included methylchavicol, spathulenol, bornyl acetate, γ-catenin, β-caryophyllene, α-limonene, and α-cadinol. The GC-MS analysis of the EO of 14 different *A. foeniculum* species from Canada prairies detected over 50 compounds, and methylchavicol was the major constituent (96 ± 2%) [42]. Skakovskii et al. [43] reported the composition rich in limonene, isomenthone, pulegone, menthone methylchavicol, and methyleugenol of the EO from *A. rugosa* (Fish. et Mey) O. Kuntze, which was determined by using ^1^H and ^13^C NMR analysis. Ivanov et al. [22] identified by GC-MS in the EO of *A. foeniculum* eight main components: estragole, eugenol, methyl isoeugenol, sylvestrene, 1-octen-3-ol acetate, β-caryophyllene, spathulenol, and caryophyllene oxide. In another study, a total of 37 components of the EO obtained from aerial parts of *A. rugosa* were determined by GC-FID and GC-MS, and the main identified compounds were methyleugenol, estragole, eugenol, thymol, pulegone, limonene, and caryophyllene [37].

In the present study, the phenolic acids and flavonoids compounds from the alcoholic plant extracts were evaluated by HPLC-DAD analysis. Phenolic compounds accumulate in various plant tissues and cells during ontogenesis and, respectively, under the influence of various environmental stimuli, being involved in many interactions of plants with their biotic and abiotic environment [44]. The chromatographic analysis of the extracts showed that the methanolic and ethanolic extracts revealed high quantities of quercetin and genistein. In the MeOH extract, the concentration of all compounds detected was higher compared to EtOH extracts, these results being similar to others from the literature [45]. Genistein and quercetin were found in high concentrations in both extracts, while tannic acid was quantifiable only in the MeOH extract. Caffeic acid, *p*-coumaric acid, quercetin, hyperoside, and rutin were identified in both extracts. Overall, the results of all analyzes confirmed that the methanolic extract presented a richer chemical composition and better biological activities than the ethanolic extract. The main compounds in the chemical composition of the aqueous and organic extracts of various *Agastache* species presented in other several studies were flavonoids (hesperitin, quercetin, tilianin), flavones (acacetin, 7-*O*-glucosyl acacetin, diosmetin 7-*O*-β-d-(6″-*O*-malonyl)-glucoside, luteolin 7-*O*-β-d-glucoside), terpenes (limonene, linalool, eugenol, ursolic acid, oleanolic acid, estragole, β-amirin), organic acids (malic acid, butanoic acid, hexadecanoic acid), esters (butanoic acid-hexane-dioctyl ester, hexanedioc-dioctyl ester, 6-octen-1-ol-3,7-dimethyl propionate, ethyl palmitate) [46]. At the flowering stage of *Agastache* species, two glucosylflavones, namely isoagastachoside and agastachin, were detected in the chemical composition of their methanolic extract [30]. Strilbytska et al. [26] detected in *A. foeniculum* leaf water extracts by HPLC-MS analysis the presence of 24 compounds with various previously described biological properties such as antioxidant, neuroprotective, antidiabetic, antineoplastic, or cardioprotective. Among these compounds, genistein and caffeic acid were also identified in our present study.

Our results showed a significant correlation between the total polyphenol and flavonoid contents (r = 0.90, *p* < 0.05) and the ability to inhibit the DPPH^•^ (r ≥ 0.93, *p* < 0.05) and ABTS^•+^ (r ≥ 0.97, *p* < 0.01) free radicals, while the power to reduce Fe^3+^ to Fe^2+^ demonstrated a good correlation with the total flavonoid contents (r = 0.93, *p* < 0.05) and DPPH^•^ radical inhibition activity (r = 0.90, *p* < 0.05). The correlations are similar to others from the literature [47,48]. The increased activity of reducing free radicals by natural compounds also justifies the results of this study in which extracts from *A. foeniculum* AdB with a high total content of flavonoids showed high antioxidant activities. The cyclic voltammetry studies demonstrate that the electrochemical oxidation of the *A. foeniculum* AdB samples is strongly related to the structure of the electroactive chemical compounds. The observed electrochemical process supports the good antioxidant activity of the EO from *A. foeniculum* AdB. Several studies have demonstrated a relationship between the high concentration of flavonoids and modulating cellular redox homeostasis processes such as ROS scavenging [6,49]. The plant extracts and EO from this study demonstrated antioxidant effects that may justify and encourage some of their uses for several disease prevention and further clinical studies.

The potential biological activities of *Agastache* species differ between subspecies, as each has a varied chemical profile. The variation in the composition of EOs and extracts of *Agastache* medicinal plants occurs due to their genetic variations, the stages of plant growth, geo-climatic conditions, nitrogen fertilizers, irrigation regimes, and maturity stage at the time of collection [36]. Due to their bioactive components, *Agastache* species could be promising therapeutic agents for human health with antioxidant, anti-inflammatory, analgesic, antimicrobial, antihypertensive, vasorelaxant, antiviral, nutraceutical, anticancer, and anti-diabetic properties [22,23,46].

In the search for new XOIs, it was shown in the current study that the extracts and EO from *A. foeniculum*, at low concentrations, exerted XO inhibition efficacy similar to allopurinol, a well-known XO inhibitor [10]. The main compound from alcoholic extracts that demonstrated XO inhibitory activities in other studies was quercetin. Flavonoids, phenols, and their glycosides have been presented in other studies to show inhibitory effects on XO [7,50,51]. These types of chemical constituents can also have an active role in the XO inhibitory effects of plant extracts in vivo due to their multiple-targets properties.

The cytotoxic activity of the EO from *A. foeniculum* AdB was evaluated in the MCF-7 breast cancer cells and the normal fibroblasts, HDF. The EO consists of multiple compounds and each compound potentially enhances or modifies the effects of others [52]. This study used the EO with all its constituents, a complex phytochemical mixture, to maximize the potential anticancer effect and to properly assess the potential risks to healthy cells and tissues. Our studies with the EO from the Romanian *A. foeniculum* AdB on MCF-7 breast cancer cell lines indicated a high cytotoxic effect at low concentrations (<0.5 µg/mL) which means that a plethora of biologically active components contributed to the high cytotoxic effect observed. With the HDF cells only at concentrations > 0.5 µg/mL, loss of viability was observed, but not as extensively as that observed for the cancer cells. Other studies reported that agastinol and agastenol, lignans detected in *Agastache* species whole plant extract, inhibited etoposide-induced apoptosis in U937 leukemia cells [29]. Estragole, the main component of the EO, has demonstrated muscle relaxant, anticonvulsant, anesthetic, bradycardic, vasoactive, anti-inflammatory, antioxidant, anticancer, and antimicrobial properties [53]. In humans, estragole usually enters the body as a component of herbal teas or as a food that has been seasoned with herbs that contain many other substances, such as flavonoids or anethole, that have a protective roles and thus reduce the possible harmful effects of pure estragole. The European Union has established maximum levels for estragole, as well as other naturally occurring compounds in plants such as methyl eugenol and safrole, in finished foods that have been flavored with flavors and/or food ingredients in which these constituents naturally occur [54]. According to the FEMA (Flavor and Extract Manufacturers Association) expert panel, these flavors continue to meet the criteria for FEMA GRAS (“generally recognized as safe”) [55]. Experimentally, estragole showed no toxicity in mice that consumed food containing this compound in low doses but possibly hepatocarcinogenic in animal experiments at high doses when given as a pure compound [56,57]. This perspective also increases when recent studies are taken into account, showing that long-term bone administration of 100 mg/kg estragole had no toxic effects in mice, probably because at low exposures they are preferentially detoxicated by biotransformation of ring substituents [53,56]. Anise hyssop EO showed a strong antioxidant capacity and also antimicrobial activity against *Staphylococcus aureus*, *Curtobacterium flaccumfaciens*, *Listeria monocytogenes*, *Bacillus subtilis*, *Salmonella* species., *Escherichia coli*, and *Pneumonia vulicans* [21]. The EO from *A. foeniculum*, rich in methyl chavicol, 1,8-cineole, 1-octen-3-ol, 3-octanone, and germacrene D, showed toxicity against two important coleopteran pests (*Oryzaephilus surinamensis* L. and *Lasioderma serricorne* F.) of stored-food products [58].

These results suggest that the extracts and EO from *A. foeniculum* may have several biological activities due to their chemical composition. This study intends to provide new contributions to the pharmacologically relevant effects of the alcoholic extracts and EO from *A. foeniculum* such as antioxidant and xanthine oxidase inhibitory activities, as well as the cytotoxic properties of the EO on cancer cells, which can be considered as evidence of the effectiveness of this medicinal plant.

## 4. Materials and Methods

### 4.1. General

All reagents were purchased from Merck (Merck KGaA, Darmstadt, Germany). The reference standards were purchased from Merck (Merck KGaA, Darmstadt, Germany), and Fluka (Honeywell Fluka™, Fluka, Germany).

Plant material. *Agastache foeniculum* (Pursh) Kuntze “Aromat de Buzău” (also known as *Lophantus anisatus*) was obtained from the Vegetable Research and Development Station in Buzău, Romania. Dr. Costel Vînătoru (Plant Genetic Resources Bank for Vegetables, Floriculture, Aromatic and Medicinal Plants, Buzău, Romania) proposed the acclimatization of *A. foeniculum* and purchased seeds belonging to this species from several sources, the main companies being Agrosemens (Z.A. du Verdalaï, 105 rue du chemin de fer, 13790 ROUSSET, Peynier, France) and Rühlemann (Rühlemann’s Kräuter & Duftpflanzen, Auf dem Berg 2, 27367, Horstedt, Germany). From a large number of assessed genotypes, after the evaluation, one of these proved great adaptability and was genetically stabilized after breeding. The selected source of seed procurement, from an original genotype of North-American origin, was Rühlemann (https://www.kraeuter-und-duftpflanzen.de/pdf/Ruehlemanns-Kraeuterkatalog-2022.pdf, accessed on 20 December 2022). After completing the acclimatization and breeding, the research was completed with the obtaining of the *A.foeniculum* “Aromat de Buzau” variety. At the same time, the cultivation of the other varieties was stopped, knowing that this one is entomophilous and can easily lose its authenticity. This new plant variety was adapted to the climatic conditions in Romania and successfully passed the testing stage for homologation and patenting in the Official Catalog of cultivated plant varieties in Romania from 2018 with plant variety ISTIS (State Institute for Testing and Registration of Varieties) patent no. 00536 by the Vegetable Research and Development Station in Buzău, Romania (https://istis.ro/en/catalog-oficial/, accessed on 15 September 2022).

**Figure 10:**
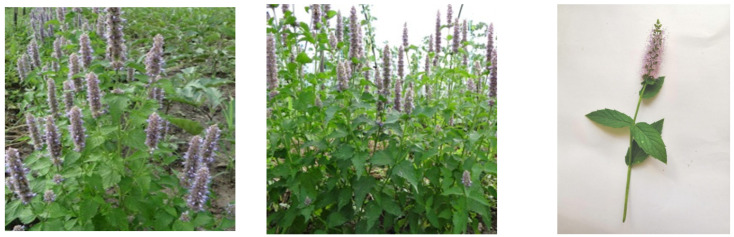
*A. foeniculum* AdB plant used in the present study.

For this study, the plants (stems, leaves, flowers) were harvested at the flowering stage in August 2020 and were further used for this research (Figure 10). A voucher specimen (ISTIS no. 00536) was deposited at the Vegetable Research and Development Station in Buzău, Romania.

Confocal laser scanning microscopy was used in this study to characterize plant cells and tissues. This analysis was performed with the Zeiss A system LSM 710 (Carl Zeiss Microscopy GmbH, 07740 Jena, Germany) equipped with a diode laser (405 nm), Ar-laser (458, 488, 514 nm), DPSS laser (diode pumped solid state e 561 nm), and HeNe-laser (633 nm). Glass microscopic slides with *A. foeniculum* AdB stem and leaf sections were observed using a Zeiss AxioObserver Z1 inverted microscope equipped with 40× apochromatic objective (1.4 aperture) and FS38, FS15, and FS49 filters. The image acquisition was made with the following parameters: in-line scan mode, average method, average number 4, speed 6, and 12-bit depth. The images of *A. foeniculum* plant cells were analyzed with ZEN 2012 SP1 software (Black Edition).

### 4.2. General Experimental Procedures

#### 4.2.1. Essential Oil Extraction

The air-dried stem, leaves, and flowers were ground to a fine powder with a grinder (Heinner, Grinder Optim150, 150 W, 50/60 Hz, Shenzhen, China). Powdered plant material (50 g) was subjected to hydrodistillation with Clevenger-type apparatus for 180 min. The distillate was extracted with diethyl ether and dried over anhydrous sodium sulfate. The EO was then stored in sealed brown vials at −20 °C before chemical analysis. The results were presented by considering three independent extraction procedures (*n* = 3).

#### 4.2.2. Gas Chromatography-Mass Spectrometry (GC-MS)

The separation and identification of volatile organic compounds (VOCs), from the analyzed oil samples, were performed by using a Trace GC Ultra Gas Chromatograph from Thermo Scientific (Thermo Fisher Scientific Inc., Bremen, Germany) coupled with Thermo Electron Polaris Q Mass Spectrometer, equipped with an electron ionization source operating at 70 eV. For the chromatographic separation of the target compounds, a DB-5MS (30 m × 0.25 mm ID, 0.25 μm film thickness) capillary column (5% diphenyl, 95% dimethylpolysiloxane) was used. The injection volume of each diluted oil sample was 2 μL. The oven temperature program was 40 °C for 3 min, which was then increased from 40 to 300 °C with 10 °C min^−1^, and kept at 300 °C for 10 min. The injector and transfer line were 250 °C and 300 °C, respectively. The carrier gas was helium with a flow rate of 1.5 mL min^−1^. The constituents of the samples were identified and confirmed based on two different approaches: linear retention indices (LRIs) determined relative to a series of n-alkanes (C8–C20) and by comparison of the mass spectra with those available in the commercial libraries (NIST 2011) [59].

#### 4.2.3. Ultrasound-Assisted Extraction with Solvents (Methanol, Ethanol)

The air-dried stem, leaves, and flowers were ground to a fine powder with a grinder (Heinner, Grinder Optim150, 150 W, 50/60 Hz, Shenzhen, China). For the extraction of phytochemical compounds, methanol and ethanol were used as solvents, and to obtain a maximum yield, ultrasound-assisted extraction was used. The powdered plant material (10 g) was extracted in alcoholic solvents (100 mL) for 120 min in a temperature-controlled ultrasonic bath (40–50 °C, Bandelin Sonorex Ultrasonic, Bandelin, Berlin, Germany, operating frequency 35 kHz, with digital timer and temperature control). The extracts were filtered through Whatman No. 4 filter paper, evaporated under a vacuum to dryness by using a rotary evaporator (RE100-Pro, DLAB Scientific Inc., Riverside, CA 92501, USA), and stored at 4 °C until analyzed. The extraction yields were determined as below [60]:

Extraction yield (%) = weight of extract (g)/weight of plant material (g) × 100(1)

#### 4.2.4. Total Phenolic Content

Total phenolic content was determined according to the literature with Folin–Ciocalteu reagent [61]. Gallic acid was used as a reference standard, and the results were expressed as milligram gallic acid equivalents per g dry weight of extract (mg GAE/g DW). All experiments were performed in triplicate. All data are expressed as the mean ± standard deviation.

#### 4.2.5. Total Flavonoid Content

Total phenolic content was determined according to the literature with 10% AlCl_3_ [61,62]. Quercetin was used as a reference standard, and the results were expressed as milligram quercetin equivalents per g dry weight of extract (mg QE/g DW). All experiments were performed in triplicate. All data are expressed as the mean ± standard deviation.

#### 4.2.6. Profiling of Bioactive Compounds by HPLC-DAD

High-performance liquid chromatography coupled with a diode array detector (HPLC-DAD) analysis was performed using an L-3000 high-performance liquid chromatography system (Rigol Technologies, INC, Beijing, China). In the chromatographic analysis, the Kinetex EVO C18 column (150 × 4.6 mm, 5 µm particle size) with an injection volume of 10 µL was used.

The extracts were analyzed after solubilizing 10 mg of each dry extract in methanol/water (80:20, *v*/*v*) and the phenolic compounds were separated on an EVO C_18_ column (150 × 4.6 mm, 5 µm particle size), at a temperature of 30 °C at 1 mL/min, with a linear gradient consisting of water-trifluoroacetic acid (99.9:0.1, *v*/*v*) and acetonitrile/trifluoroacetic acid (99.9:0.1, *v*/*v*) [63]. The standards used for HPLC-DAD: *p*-coumaric acid CAS No: 501-98-4, caffeic acid CAS No: 331-39-5, rutin CAS No.: 153-18-4, quercetin CAS No.: 849061-97-8, hyperoside CAS No.: 482-36-0, tannic acid CAS No.: 1401-55-4, genistein CAS No.: 446-72-0, and naringenin CAS No.: 67604-48-2, were purchased from Sigma-Aldrich (Merck KGaA, Darmstadt, Germany), dissolved in methanol/water, and used in HPLC analysis according to previous works [62,64,65]. The phenolic compounds were identified by combining the following information: elution order, retention time in the C_18_ column, comparison with standard solutions analyzed under the same conditions, and quantified by using analytical curves of the analyzed compounds [62,64]. The compound contents were expressed as mg/g of dried weight, considering three independent extraction procedures (*n* = 3).

### 4.3. Antioxidant Assays

#### 4.3.1. In Vitro-Antioxidant Assays

In the present study, four methods were used to evaluate the antioxidant activity of the alcoholic extracts and EO from *A. foeniculum* AdB. For each assay, standard compounds were used as positive controls. All details of the antioxidant assays were published in previous studies [61,64,66]. All the analyses were performed in triplicate, and the results were reported as mean ± SD.

The antioxidant activity of the extract was determined by the DPPH assay, as previously described [61]. Briefly, 100 μL of each extract (2–1000 µg/mL) was mixed with 100 µL DPPH solution and incubated in the dark at room temperature for 1 h. The absorbance of the mixture was measured at 517 nm with a microplate reader with 96-well plates (Tecan Pro 200, Tecan Trading AG, Männedorf, Switzerland). Gallic acid is known for its antioxidant potential and was used as a positive control [67]. The ability of the sample to scavenge DPPH radical was determined with the following equation:

DPPH scavenging effect = (OD_Control_ − OD_Sample_)/OD_Control_ × 100 (2)

where OD_Control_ represents the absorbance of the control, and OD_Sample_ represents the absorbance of the sample.

The scavenging activity of each sample against 2,2′-azinobis-3-ethylbenzothiazoline-6-sulfonic acid radical was carried out according to the Busuioc et al. method [64]. Trolox was used as a positive antioxidant reference control [65]. The ABTS radical cation was produced by the reaction between ABTS solution (7 mM) and K_2_S_2_O_8_ aqueous solution (15 mM) for 12 h, in dark conditions at room temperature. Then, the ABTS radical cation solution was diluted with deionized water to obtain an absorbance of 0.70 ± 0.02 at 734 nm. The ABTS radical cation solution (100 µL) was added to 100 µL extract solution of various concentrations (0.25, 0.5, 1.0, 1.5, 2.0, 2.5, and 3.0 mg/mL). After each 15 min of incubation in dark conditions, the absorbance at 734 nm was determined with a microplate reader with 96-well plates (Tecan Pro 200, Tecan Trading AG, Männedorf, Switzerland). The scavenging activity of the samples against radical ABTS was evaluated by the following Equation (3):

ABTS scavenging activity = (OD_Control_ − OD_Sample_)/OD_Control_ × 100 (3)

where OD_Control_ represents the absorbance of the control, and OD_Sample_ represents the absorbance of the sample.

The IC50 values were determined from the relationship curve of radical scavenging activity versus concentrations of the respective sample curve. A lower IC50 value means higher antioxidant activity. The IC50 value is a parameter commonly used to measure the antioxidant activity of various samples. It is evaluated as the concentration of antioxidants necessary to decrease the initial DPPH concentration by 50% [68].

Determination of antioxidant activity by FRAP assay was carried out according to the procedure previously described in the literature [65], by monitoring the reduction of Fe^3+^-tripyridyl triazine (TPTZ) to blue-colored Fe^2+^-TPTZ measured at 593 nm. The FRAP reagent was freshly prepared by adding a 10:1:1 (*v/v/v*) ratio of 0.3 M acetate buffer (pH 3.6), 10 mM TPTZ in 40 mM HCl, and 20 mM FeCl_3_. The assay was performed by mixing 50 μL of sample in a 96-well microplate and then adding 200 μL of FRAP reagent. After 15 min of incubation and shaking at 37 °C, the absorbance was read at 593 nm with a microplate reader with 96-well plates (Tecan Pro 200, Tecan Trading AG, Männedorf, Switzerland). FRAP was expressed as µM FeSO_4_ equivalents/g of extract. All samples were analyzed in triplicate.

#### 4.3.2. Electrochemical Evaluation of Antioxidant Capacity by Cyclic Voltammetry

The EO from *A. foeniculum* AdB was electrochemically evaluated for its antioxidant potential by using SP-150 Biologic potentiostat/galvanostat (Bio-Logic SAS, Claix, France). The experiments were conducted at room temperature with an electrochemical cell equipped with three electrodes: platinum wire (counter electrode), Ag/AgCl_sat_ (E = 0.194 V/NHE) (reference electrode), and glass carbon electrode (working electrode). For this assay, the applied potential was E = ±2 V vs. Ag/AgCl_sat_ and the scan rate was set at 100 mVs^−1^. The working electrode was polished with alumina and diamond slurries (BASi^®^ polishing kit), followed by cleaning with ethanol after each measurement. All measurements were conducted in triplicate.

### 4.4. Xanthine Oxidase Inhibitory Activity Assays

This assay was established as previously described [9], with a few modifications, and adapted to the microplate. This method is based on monitoring uric acid formation in the xanthine oxidase system [9]. Freshly prepared substrate (0.6 mM xanthine dissolved in 0.1 N NaOH), and enzymatic (0.25 XO units/mL in 0.1 M phosphate buffer, pH 7.5) solutions were used in this assay. The EO from *A. foeniculum* AdB of various concentrations was dissolved in 1% DMSO in phosphate buffer (pH 7.5). The extracts were dissolved in phosphate buffer at various concentrations. The assay mixture consisted of 40 µL of test solution, with 40 µL phosphate buffer (pH 7.5), 20 µL of enzyme solution (0.25 U/mL), and xanthine solution (100 µL). The reaction was initiated by adding the enzyme with or without inhibitors. Allopurinol was used as a positive control because it is a well-known XO inhibitor [10]. Changes in the absorbance of the mixtures were spectrophotometrically determined at 295 nm with a microplate spectrophotometer (Tecan Pro 200, Tecan Trading AG, Männedorf, Switzerland), at 30 s intervals for 3 min, and 25 °C. Determination of the inhibition activity (I) was made by the following Equation (4):

% inhibition = (OD_Control_ − OD_Sample_)/OD_Control_ × 100 (4)

where OD_Control_ represents the absorbance of the control sample without inhibitor, and OD_Sample_ represents the absorbance of the sample.

The results were statistically analyzed by comparing the xanthine oxidase inhibition values obtained at baseline (control) with those obtained in different treatments by analysis of variance (ANOVA) followed by the Dunnett test (multiple comparisons with one control) with *p* < 0.05.

### 4.5. Cytotoxic Activities

The effect of the EO from *A. foeniculum* AdB on the cellular viability of MCF-7 (ATCC) and HDF (Sigma-Aldrich) cell lines was measured using the MTT assay, as previously reported [38]. Cells were grown in DMEM + Glutamax I medium (MCF-7) supplemented with 10% FBS and 1% antibiotics or fibroblast growth medium (HDF) without supplements. For the assays, cells were seeded in 96-well plates and left for 24 h to adhere in a 5% CO_2_ incubator at 37 °C. After the media was removed and 200 µL of complete media with serial dilutions of the *A. foeniculum* EO were applied to each well. After 24 h of incubation, the supernatant was removed, MTT (0.5 mg/mL PBS) was added, and the plates were incubated for 3 h at 37 °C. The purple formazan formed was dissolved in 200 µL DMSO. Optical density was measured at 570 nm using a microplate reader (Power Wave Xs, Bio-Tek, Winooski, VT, USA). The results were statistically analyzed by comparing the HDF and MCF-7 values obtained at baseline (control) with those obtained in different treatments by analysis of variance (ANOVA) followed by the Dunnett test (multiple comparisons with one control) with *p* < 0.05.

### 4.6. Statistical Analysis

The experiments were performed in triplicate, and the data presented represent the average of the three determinations ± standard deviation (SD). The IC_50_ values were found using the GraphPad Prism software (vs. 5.0). The data were statistically analyzed using IBM SPSS Statistics software (version 29, IBM Corp., New York, NY, USA). Statistical evaluation of the obtained data was assessed by one-way analysis of variance (ANOVA) followed by the Duncan multiple range test to find out significant differences (*p* ≤ 0.05). Pearson’s correlation coefficients were computed to identify the relationship between antioxidant activities, total polyphenols, and flavonoid contents, and a statistical correlation was considered significant at the 0.05 level (*p* < 0.05). The results from the biological assays were statistically analyzed by analysis of variance (ANOVA) followed by the Dunnett test (multiple comparisons with one control) to find out significant differences (*p* ≤ 0.05) among the obtained data.

## 5. Conclusions

This study is the first to investigate the phytochemical profiling and in vitro biological properties of the EO and alcoholic extracts from the *A. foeniculum* “Aromat de Buzau” variety. The EO and alcoholic extracts showed good antioxidant properties and inhibitory potential against xanthine oxidase, a critical enzyme involved in pathologies like gout. The EO also revealed selective cytotoxic activity against breast cancer MCF-7 cells compared to normal HDF fibroblasts; however, further in vivo and clinical studies are needed. This plant is a source of bioactive compounds that could be good candidates in therapeutic applications to treat human diseases and may find use as raw material for the nutraceutical, food, pharmaceutical, and cosmetic industries, and may be synergistically used with other antioxidants and chemotherapeutic agents.

## Figures and Tables

**Table 1:** The chemical composition of the EO from *A. foeniculum* AdB.

No.	Rt (min.)	Constituent	m/z	Molecular Formula & Molecular Weight	Concentration (%)
1	8.16	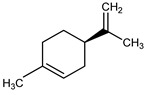 Limonene	136	C_10_H_16_(MW: 136.23)	2.91 ± 0.65 ^b^
2	10.82	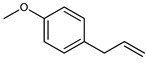 Methyl chavicol (Estragole)	148	C_10_H_12_O(MW: 148.20)	94.89 ± 1.02 ^a^
3	12.07	Unknown			0.40 ± 0.28 ^d^
4	12.76	Unknown			0.04 ± 0.02 ^f^
5	12.97	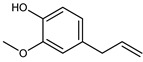 Eugenol	164	C_10_H_12_O_2_(MW: 164.20)	0.01 ± 0.01 ^g^
6	13.18	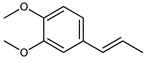 Methyl isoeugenol	178	C_11_H_14_O_2_(MW: 178.23)	0.04 ± 0.01 ^f^
7	13.57	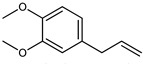 Methyl eugenol	178	C_11_H_14_O_2_(MW: 178.23)	0.73 ± 0.04 ^c^
8	13.98	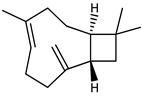 Caryophyllene	204	C_15_H_24_(MW: 204.35)	0.74 ± 0.02 ^c^
9	14.75	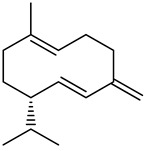 Germacrene D	204	C_15_H_24_(MW: 204.35)	0.23 ± 07 ^e^

The values followed by the same letter (a, b, c, d, e, f, g) in the same column show no statistically significant differences (*p* < 0.05) according to the analysis of variance (ANOVA). Each value is the mean of three replicates ± standard deviation (SD).

**Table 2:** Total phenolic and flavonoid contents of methanolic and ethanolic extracts of *A. foeniculum* AdB; means ± SD.

Samples	Extraction Yield (%)	TPC (mg GAE/g DW)	TFC (mg QE/g DW)
MeOH	11.062 ± 0.945 ^a^	485.084 ± 0.052 ^a^	367.32 ± 0.008 ^a^
EtOH	7.211 ± 0.686 ^b^	403.918 ± 0.057 ^b^	355.94 ± 0.007 ^b^

TPC: total polyphenol content; TFC: total flavonoid content; MeOH: methanolic extract; EtOH: ethanolic extract; GAE: gallic acid equivalents; DW: dry weight; QE: quercetin equivalents. The values followed by different letters (a, b) in the same column show statistically significant differences (*p* > 0.05). Each value is the mean of three replicates ± SD.

**Table 3:** Bioactive compounds contained in ethanolic (EtOH) and methanolic (MeOH) extracts of *A. foeniculum* AdB.

Chemical Name	Chemical Structure	Content (µg/g dw)	
		MeOH	EtOH
**Phenolic Acids**			
*p*-Coumaric acid	C_9_H_8_O_3_	62.079 ± 0.436 ^a^	8.982 ± 0.093 ^b^
Caffeic acid	C_9_H_8_O_4_	59.014 ± 0.103 ^a^	31.320 ± 0.145 ^b^
**Tannins**			
Tannic acid	C_76_H_52_O_46_	72.061 ± 0.077 ^a^	-
**Flavonols**			
Rutin	C_27_H_30_O_16_	76.409 ± 0.100 ^a^	56.701 ± 0.111 ^b^
Quercetin	C_15_H_10_O_7_	1073.637 ± 0.130 ^a^	704.148 ± 0.150 ^b^
Hyperoside	C_21_H_20_O_12_	98.693 ± 0.190 ^a^	58.892 ± 0.105 ^b^
**Flavanones**			
Naringenin	C_15_H_12_O_5_	43.683 ± 0.114 ^a^	30.242 ± 0.121 ^a^
**Isoflavones**			
Genistein	C_15_H_10_O_5_	3171.823 ± 0.218 ^a^	2229.999 ± 0.256 ^b^

MeOH: methanolic extract; EtOH: ethanolic extract; dw: dry weight. The values followed by the same letter (a, b) in the same row show no statistically significant differences (*p* < 0.05) according to the analysis of variance (ANOVA). Each value is the mean of three replicates ± SD.

**Table 4:** IC_50_ values for antioxidant assays.

Sample	DPPH IC_50_ Value	ABTS IC_50_ Value	FRAP (µM Fe(II) Equivalents/g of Extract)	Unit of Measurement
MeOH	76.368 ± 0.002 ^b^	98.274 ± 0.002 ^b^	45.721 ± 0.014 ^a^	µg/mL
EtOH	94.986 ± 0.002 ^a^	244.261 ± 0.003 ^a^	39.483 ± 0.017 ^a^	µg/mL
EO	12.943 ± 0.001 ^c^	0.3356 ± 0.002 ^d^		µg/mL
Trolox		32.562 ± 0.002 ^c^		µM
Gallic acid	15.614 ± 0.012 ^d^			µg/mL

The values followed by the same letter (a, b, c, d) in the same column show no statistically significant differences (*p* < 0.01). Each value is the mean of three replicates ± SD.

**Table 5:** Pearson correlation matrix between phytochemical contents and antioxidant activities of *A. foeniculum* extracts.

	TPC ^a^	TFC ^b^	DPPH^•c^	ABTS^•+ d^	FRAP ^e^
TPC	1				
TFC	0.9087 *	1			
DPPH^•^	0.9373 *	0.9972 **	1		
ABTS^•+^	0.9720 **	0.9813 **	0.9929 **	1	
FRAP	0.7356	0.9275 *	0.9057 *	0.8600	1

^a^ Total phenolic content, ^b^ Total flavonoid content, ^c^ IC50 in DPPH assay, ^d^ IC50 in ABTS assay, ^e^ FRAP assay (µM Fe(II) equivalents); * Correlation is significant at the *0.05* level; ** Correlation is significant at the *0.01* level.

## Data Availability

Not applicable.

## Supplementary Materials

The following supporting information can be downloaded at: https://www.mdpi.com/article/10.3390/ijms24010828/s1.
