# Supplementary material for: Bridging the Chemical Profile and Biological Activities of a New Variety of Agastache foeniculum (Pursh) Kuntze Extracts and Essential Oil
Source: Int J Mol Sci. 2023 Jan 3;24(1):828. doi: 10.3390/ijms24010828 (PMC9821440; doi:10.3390/ijms24010828)
Supplement: Supplementary file 1 [file ijms-24-00828-s001.zip › ijms-supplementary.pdf]

International Journal of
Molecular Sciences

MDPI

Supplementary Materials:

Bridging the Chemical Profile and Biological Activities of a New Variety of *Agastache foeniculum* (Pursh) Kuntze Extracts and Essential Oil

Fănică Bălănescu [1,2], Andreea Veronica Botezatu [2,*], Fernanda Marques [3], Anna Busuioc [2], Olivian Marincaş [4], Costel Vînătoru [5], Geta Cârâc [2], Bianca Furdui [2] and Rodica Mihaela Dinica [2,*]

[1] Faculty of Medicine and Pharmacy, "Dunărea de Jos" University of Galati, 35 A.I. Cuza Street, 800010 Galati, Romania
[2] Faculty of Sciences and Environment, Department of Chemistry Physical and Environment, "Dunărea de Jos" University of Galati, 111 Domnească Street, 800201 Galati, Romania
[3] Departamento de Engenharia e Ciências Nucleares (DECN), Centro de Ciências e Tecnologias Nucleares, Instituto Superior Técnico, University of Lisbon, Campus Tecnológico e Nuclear, Estrada Nacional 10, Km 139.7, 2695-066 Bobadela, Portugal
[4] National Institute for Research and Development of Isotopic and Molecular Technologies, 67-103 Donat Street, 400293 Cluj-Napoca, Romania
[5] Plant Genetic Resources Bank for Vegetables, Floriculture, Aromatic and Medicinal Plants Buzău, 56 Nicolae Bălcescu Street, 120187 Buzau, Romania
* Correspondence: andreea.botezatu@ugal.ro (A.V.B.); rodica.dinica@ugal.ro (R.M.D.)

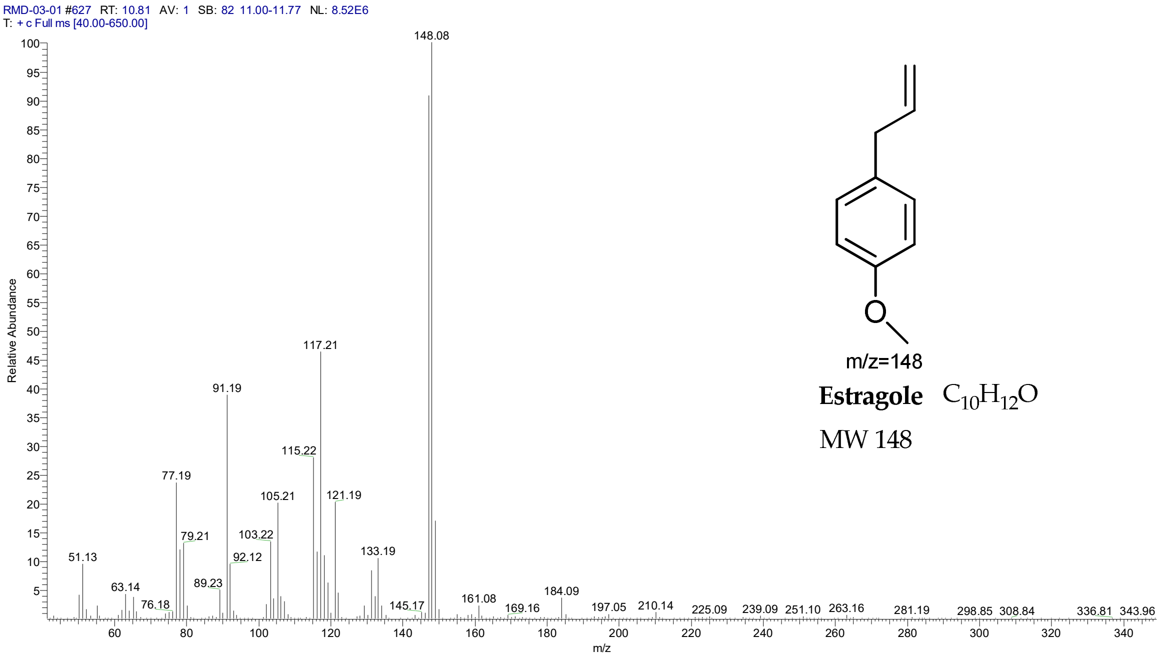

Figure S1. GC/MS spectra and fragmentation patterns of methyl chavicol (Estragole)

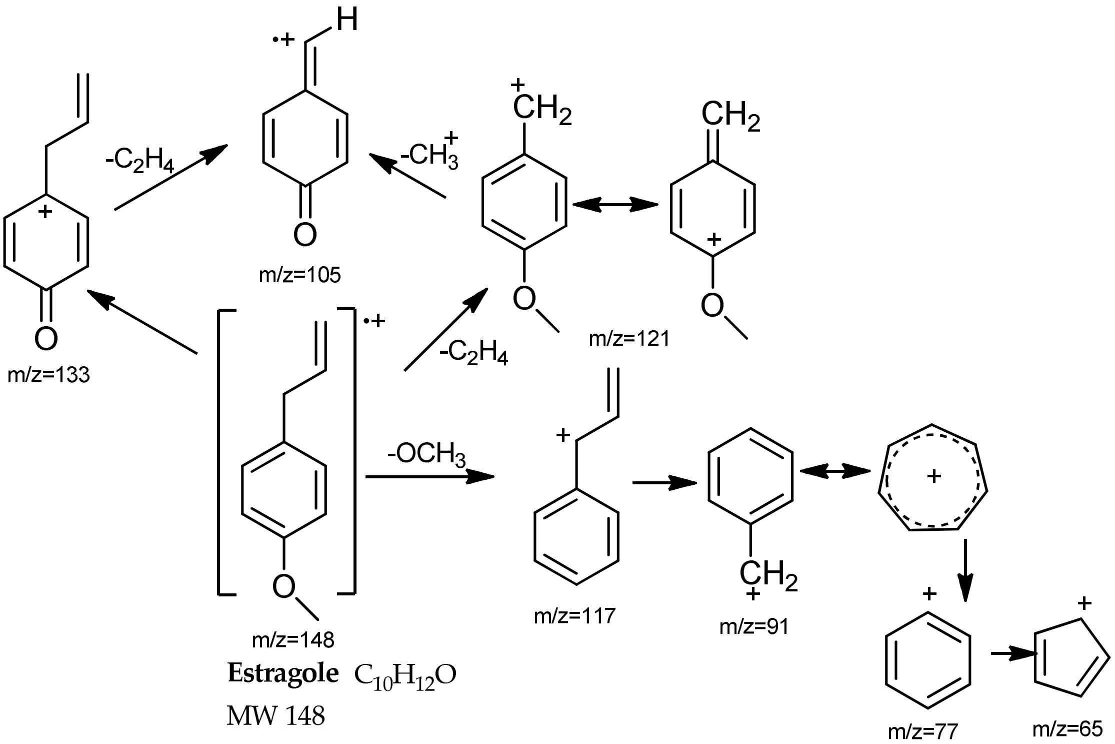

Figure S2. Mass spectrum interpretation of methyl chavicol (Estragole)

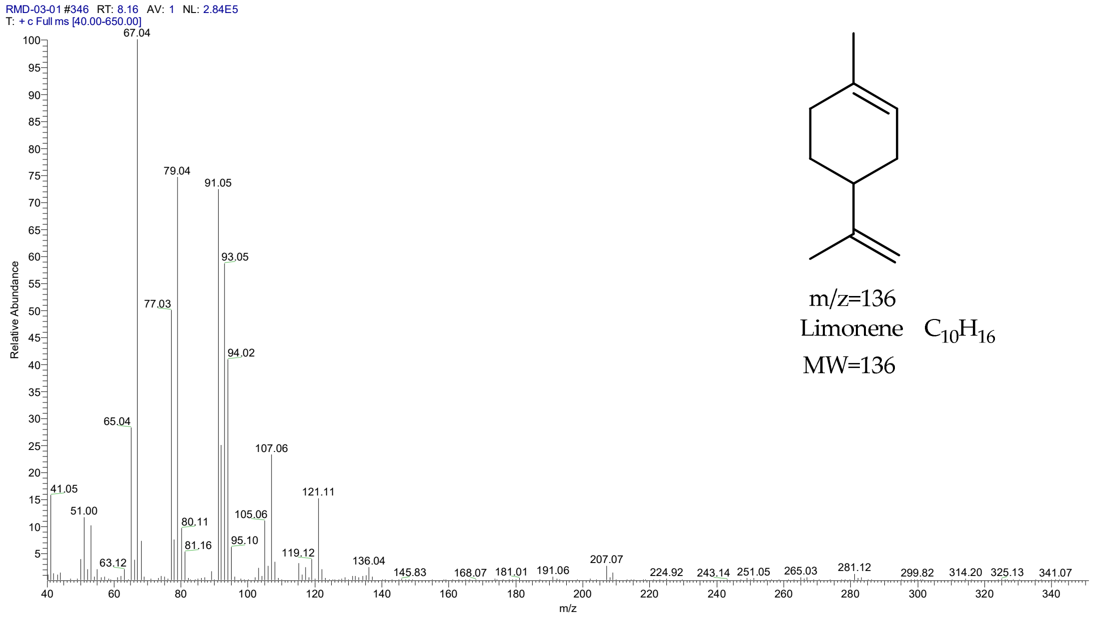

Figure S3. GC/MS spectra and fragmentation patterns of limonene

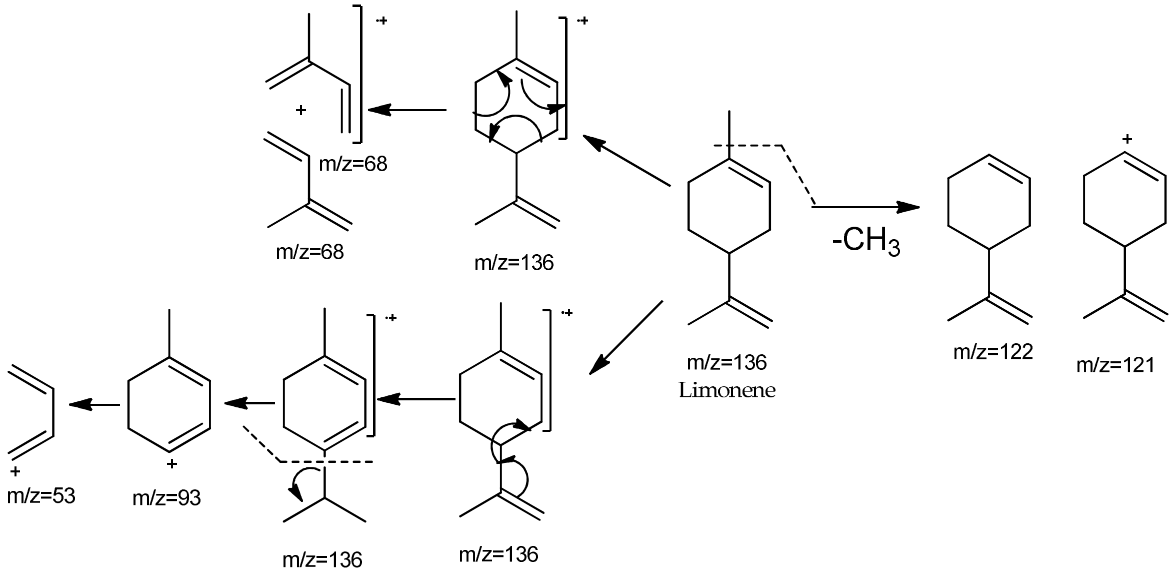

Figure S4. Mass spectrum interpretation of limonene

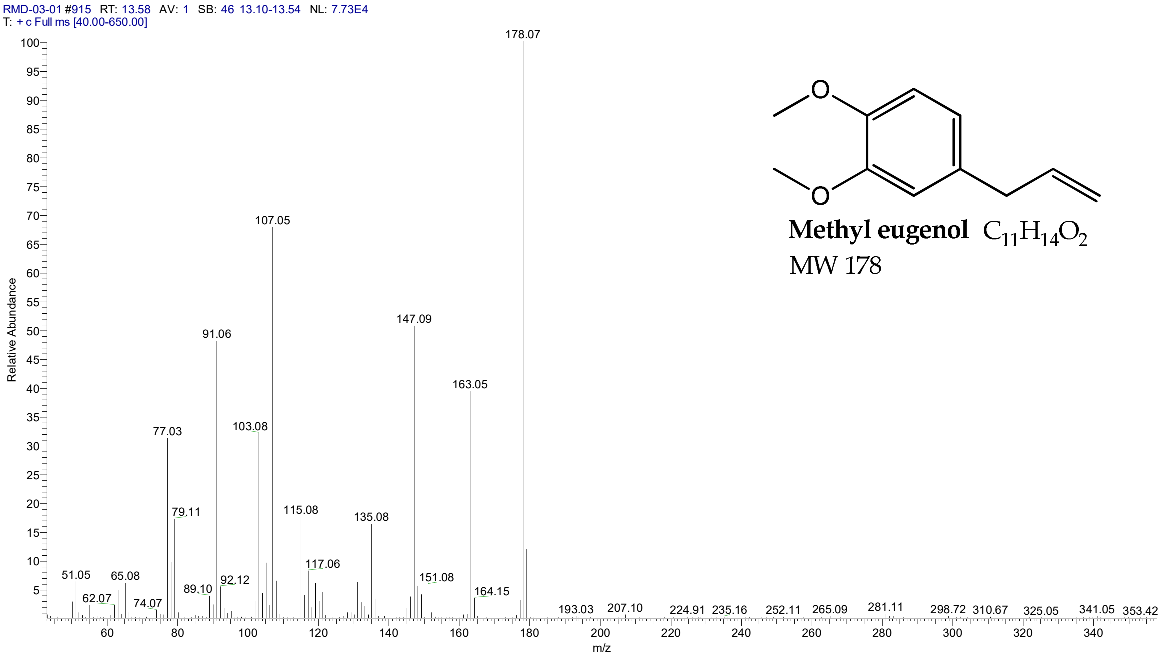

Figure S5. GC/MS spectra and fragmentation patterns of methyl eugenol

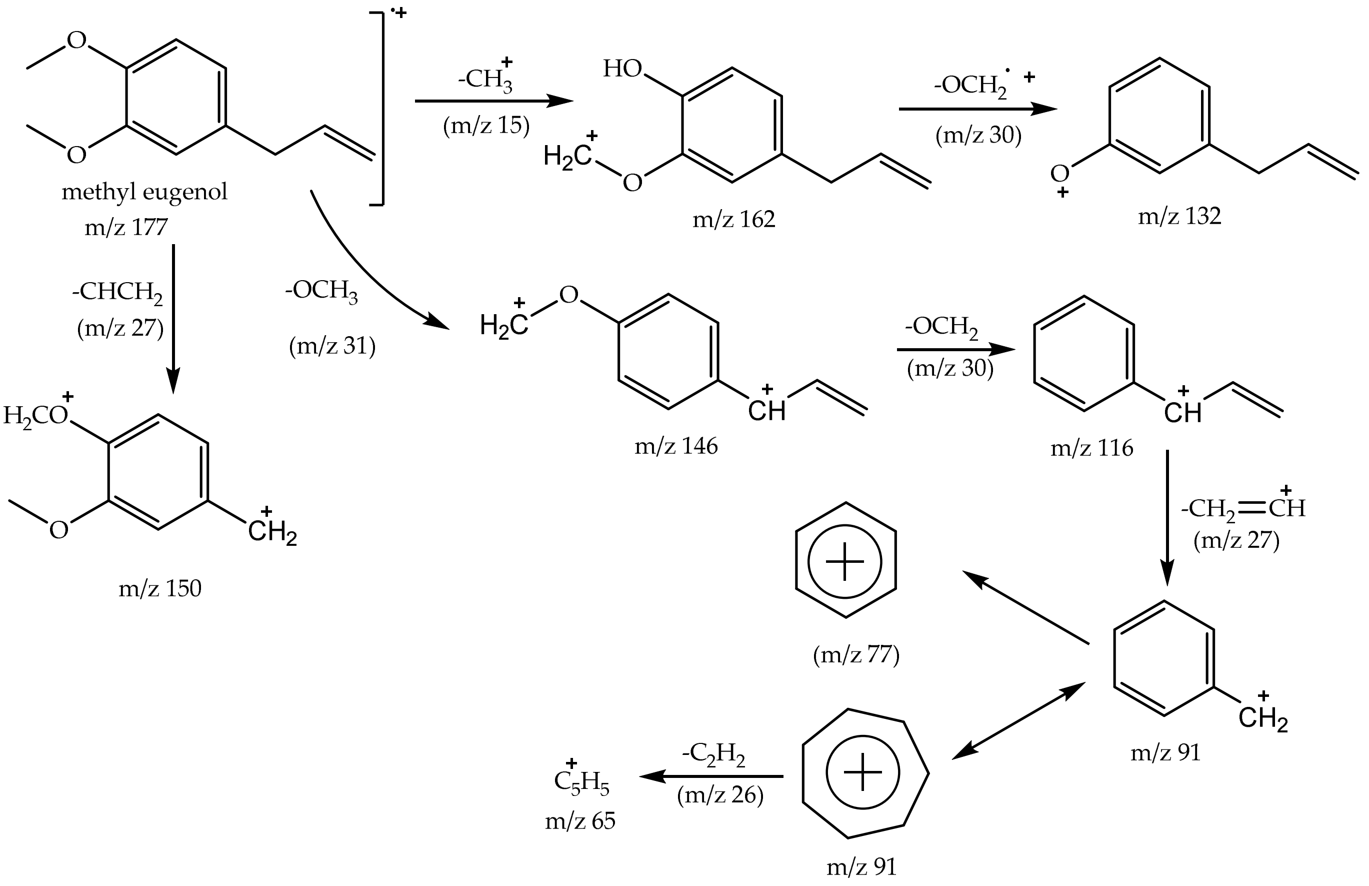

Figure S6. Mass spectrum interpretation of methyl eugenol

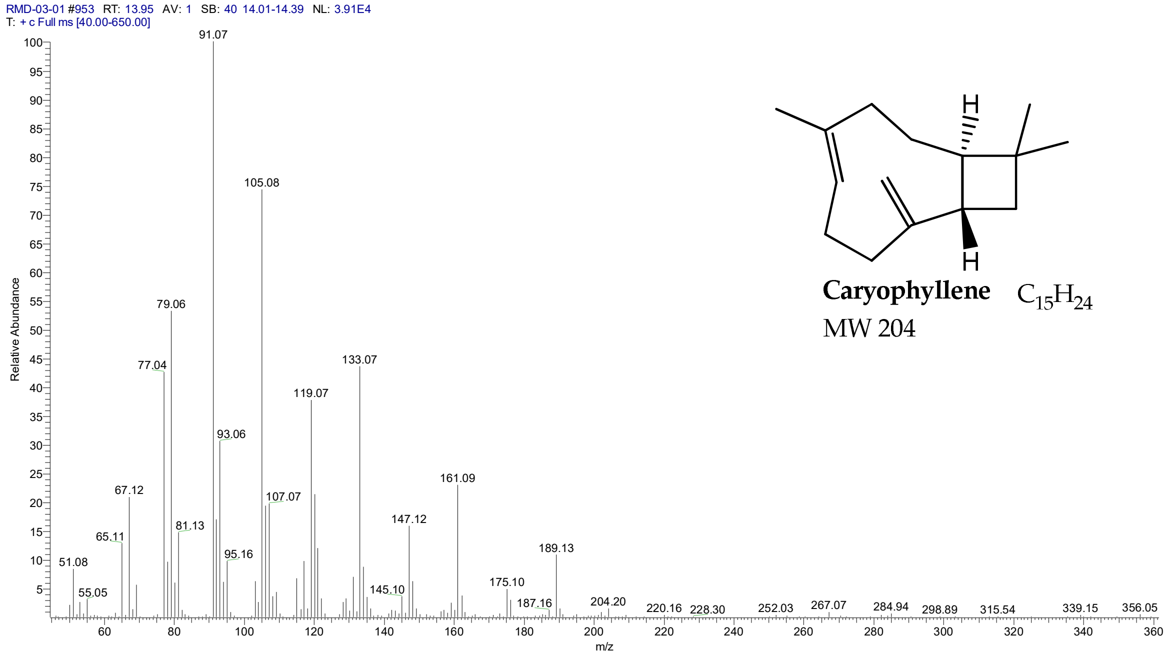

Figure S7. GC/MS spectra and fragmentation patterns of Caryophyllene

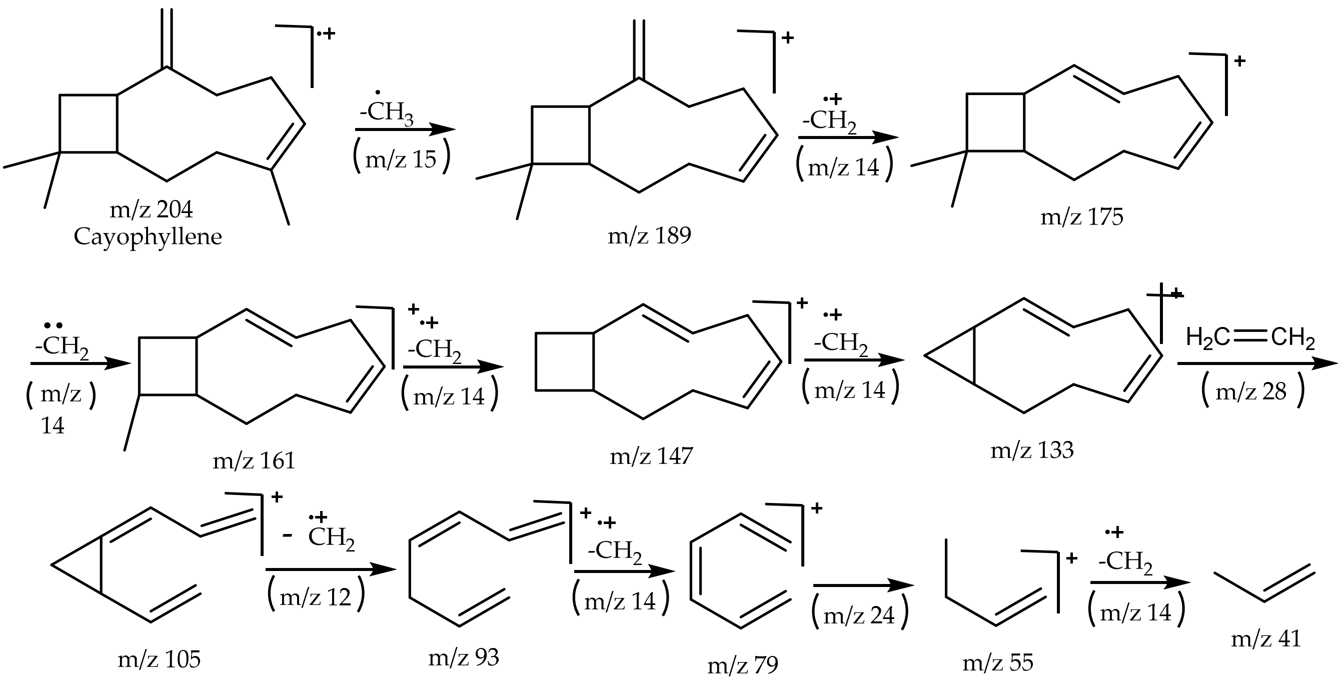

Figure S8. Mass spectrum interpretation of Caryophyllene
